# The Evolution of Cataract Extraction

**Published:** 1899-11-04

**Authors:** 


### THE EVOLUTION OF CATARACT EXTRACTION.

At the meeting of the Ophthalmological Society, held on October 19th, the President, Mr. Anderson Critchett, in his opening address gave a short account of the different changes which had occurred in the operation for removal of cataract, in the course of its evolution, since it was first attempted by Daviel in 1745. Prior to that time couching and discission were the only recognised operations. Daviel's operation was a simple extraction, and though it gained numerous adherents there were many who continued to rely on the more primitive methods, and even as late as 1838 it was stated by Guthrie that "there are reasons which will ever prevent it being practised by the great body of the profession." The statistics of the operation by extraction were, however, remarkably good, and when done with skill and success the results given by it were excellent. Unfortunately, a large prolapse of iris often occurred, and in those pre-antiseptic days primary suppurations were numerous. The dawn of a new era was foreshadowed at Moorfields when it was found that the results of one of the surgeons, who was less skilful than his fellows, were nevertheless exceptionally good. This gentleman, as well as purposely removing the lens, usually accidentally did an iridectomy. The first definite change was made by Professor Schuft, who made a slit-like opening at the corneo-scleral junction, did a large iridectomy, and then introduced a spoon with which to remove the lens. This, however, often caused much loss of vitreous. Then, in 1864, the president's late father read a paper at the congress at Heidelberg on the excellent results achieved by himself and Mr. Bowman with linear extraction, and this led to Von Graafe introducing the knife still used and always associated with his name. The operation he instituted had its dangers, and without great skill and care the operator was liable to encroach on the ciliary region, and severe cyclitis and loss of vitreous was not infrequent. Numerous modifications had since been made. Pagenstecher removed the lens with a spoon, as also did Macnamara and Andrew of Shrewsbury; Lebrun of Paris and Pridgin Teale made a purely corneal section, but anterior synechiæ and astigmatism were frequently produced. Bell Taylor removed a small portion of the periphery of the iris and left the pupil intact, but the difficulties of the operation were serious drawbacks to its adoption. Ophthalmic surgeons were now divided into two great camps; on the one side were the adherents of simple extraction, while on the other were those who were loyal to iridectomy. In spite of recent advances finality has not yet been reached.

